# A little longer, a lot better: simulation-guided exploration of extended-length single-end barcoded reads for structural variant detection

**DOI:** 10.1093/bioadv/vbag267

**Published:** 2026-09-08

**Authors:** Can Luo, Yichen Henry Liu, Han Liu, Zhenmiao Zhang, Lu Zhang, Brock A Peters, Xin Maizie Zhou

**Affiliations:** Department of Biomedical Engineering, Vanderbilt University, Nashville, TN 37235, United States; Department of Computer Science, Vanderbilt University, Nashville, TN 37235, United States; Department of Computer Science, Vanderbilt University, Nashville, TN 37235, United States; Department of Computer Science and Engineering, University of California San Diego, San Diego, CA 92093, United States; Department of Computer Science, Hong Kong Baptist University, Hong Kong, China; Advanced Genomics Technology Lab, Complete Genomics Inc, San Jose, CA 95134, United States; Department of Biomedical Engineering, Vanderbilt University, Nashville, TN 37235, United States; Department of Computer Science, Vanderbilt University, Nashville, TN 37235, United States

## Abstract

**Motivation:**

Accurate detection of genetic variants, including single nucleotide polymorphisms (SNPs), small insertions and deletions (INDELs), and structural variants (SVs), is essential for comprehensive genomic analysis. While short-read sequencing performs well for SNP and INDEL detection, it remains limited in resolving SVs, particularly in complex genomic regions, due to its short read length. Linked-read sequencing technologies, such as single-tube Long Fragment Read (stLFR), partially address this limitation by incorporating molecular barcodes to provide long-range information.

**Results:**

In this study, we evaluate conventional paired-end linked reads (PE100_stLFR) and explore a conceptual extension: long single-end barcoded reads of 500 bp (SE500_stLFR) and 1000 bp (SE1000_stLFR). We developed stLFR-sim, a Python-based simulator that reproduces the stLFR workflow and enables realistic benchmarking. Using a high-quality T2T assembly of HG002, we generated multiple datasets across 12 sequencing configurations. SVs were called using Aquila_stLFR (v2) and benchmarked against the Genome in a Bottle (GIAB) HG002 SV truth set with Truvari. We show that simulated PE100_stLFR has the same trade-off pattern between precision and recall in SV calling compared to real data. Increasing read length consistently improves SV detection accuracy, with SE1000_stLFR achieving the best performance among the evaluated stLFR configurations and showing competitive performance relative to ICLR- and pangenome-based approaches, while approaching the performance of long-read methods. Collectively, our results highlight the potential of extended-length single-end barcoded reads for improving SV detection and demonstrate how simulation can be used to evaluate prospective linked-read sequencing designs.

**Availability and implementation:**

stLFR-sim is a Python-based, open-source simulator for paired-end and extended-length single-end barcoded sequencing. The software is freely available under the MIT License at https://github.com/maiziezhoulab/stLFRsim.

## 1 Introduction

Advancements in sequencing technologies have revolutionized genomics study by enabling precise and high-throughput analysis of DNA, offering unprecedented opportunities to understand genetic variation, disease mechanisms (Ng *et al.* 2010), and evolutionary biology (Pääbo 2014). Among these advancements, short-read sequencing (Margulies *et al.* 2005, Bentley *et al.* 2008) has become the backbone of many genomic studies due to its cost-effectiveness, scalability, and high accuracy in detecting small variants, such as single nucleotide polymorphisms (SNPs) and small insertions and deletions (indels). However, despite its transformative impact, the intrinsic limitations of short-read sequencing in terms of read length impose significant challenges when addressing more complex genomic features, such as structural variants (SVs), repetitive regions, and chromosomal rearrangements. The inability to span long repetitive sequences or phase variants across distant loci restricts its utility for comprehensive genome characterization, leaving gaps in our understanding of genetic variation.

To overcome these challenges, innovative technologies have been developed to extend the capabilities of sequencing, providing long-range information and improved resolution of complex genomic features. One notable advancement is linked-read sequencing, which combines short-read sequencing with molecular barcoding to retain long-range context (Zheng *et al.* 2016, Wang *et al.* 2019). This method starts by fragmenting high molecular weight (HMW) DNA into long segments, typically tens to hundreds of kilobases in length. Each fragment is encapsulated into microfluidic droplets or physically separated into individual compartments, where it undergoes amplification and tagging with a unique molecular barcode. Importantly, all short reads derived from a single DNA fragment carry the same unique barcode, allowing them to be computationally grouped and assigned to their original long DNA molecule. Once the barcoded DNA fragments are sequenced, bioinformatics tools can reconstruct the long-range structure of the original DNA by clustering reads based on their shared barcode (Liu *et al.* 2021, Zhou *et al.* 2021, Luo *et al.* 2025). This enables linked-read sequencing to effectively span long regions of the genome, even with the relatively short read lengths produced by sequencing platforms like Illumina. This methodology enhances genome assembly, variant detection, and haplotype phasing by preserving the positional information of DNA fragments, which is critical for resolving structural variants and complex regions (Zhang *et al.* 2020, Hu *et al.* 2021, 2022a, 2022b, Wagner *et al.* 2022).

Among linked-read technologies, the 10x Genomics Chromium platform stands out as a pioneering implementation (Zheng *et al.* 2016). It uses microfluidics to partition HMW DNA into thousands of droplets, each containing a unique barcode and reagents for DNA amplification. This approach ensures high efficiency and scalability, enabling the analysis of large and complex genomes. However, one limitation of this method is the occurrence of multiple DNA molecules within a single droplet, leading to potential barcode collisions and reduced resolution in some cases. The advent of single-tube long fragment read (stLFR) technology addressed this issue by eliminating the need for droplet-based separation and ensuring a “near” one-to-one correspondence between individual DNA molecules and barcodes (Wang *et al.* 2019). Using a bead-based chemistry, stLFR introduces barcodes directly to DNA fragments in solution, simplifying the workflow and improving the accuracy of molecule-to-barcode association. This innovation further enhances the resolution of linked-read sequencing, particularly for applications requiring high precision in structural variant detection and haplotype phasing. Despite these advancements, the SV discovery performance of traditional paired-end linked reads still remains inferior to that of long-read sequencing. This led us to ask whether modest modifications, such as slightly extending the read length, combined with the power of barcode information could achieve performance approaching that of long-read technologies.

Motivated by this idea, this study explores a conceptual extension of linked-read technology: long single-end barcoded reads with lengths of 500 bp and 1000 bp. Unlike the traditional paired-end reads commonly used in linked-read sequencing, barcoded single-end reads could simplify library preparation workflows while leveraging longer individual reads per barcode to provide improved mapping resolution. This design has the potential to enhance structural variant (SV) detection, particularly in genomic regions that are difficult to resolve using conventional short-read and paired-end approaches. To evaluate this concept, we simulated different linked-read data, including conventional paired-end linked read (PE100_stLFR) and the conceptual single-end linked-read (SE500_stLFR and SE1000_stLFR) of 12 sequencing configurations (Table 1), respectively. We first compared the variant calling performance between simulated PE100_stLFR data and real PE100_stLFR data to assess whether the simulation framework captures broad variant-calling characteristics observed in real linked-read data. We then systematically assessed SV detection performance using stLFR-sim–generated libraries, including both conventional paired-end linked reads and conceptual single-end barcoded reads. Our results demonstrate that barcoded sequencing with moderately extended read lengths can substantially improve SV detection accuracy, achieving performance approaching that of long-read sequencing and pangenome-based short-read genotyping strategies. These findings suggest that if long single-end barcoded reads become technically feasible, it could provide a highly effective strategy for accurate structural variant discovery. Our work therefore provides a simulation-based framework for exploring future linked-read sequencing designs.

**Table 1:** The table summarizes the simulation settings for all experiments (datasets).

**Shared Parameters:** $N_{FL}$** = 1; Insert Size = 600 bp; Error Rate = 1%**			
	**PE100_stLFR/SE500_stLFR/SE1000_stLFR 35x**		
	$C_{F}$	$C_{R}$	$\mu_{FL}(kb)$
**EXP1**	350	0.1	50
**EXP2**	140	0.25	50
**EXP3**	70	0.5	50
**EXP4**	47	0.75	50
**EXP5**	350	0.1	75
**EXP6**	140	0.25	75
**EXP7**	70	0.5	75
**EXP8**	47	0.75	75
**EXP9**	350	0.1	100
**EXP10**	140	0.25	100
**EXP11**	70	0.5	100
**EXP12**	47	0.75	100

All datasets share the same $N_{FL}$ (number of fragments per barcode), insert size, and error rate. PE100_stLFR, SE500_stLFR, and SE1000_stLFR indicate the read length and whether sequencing is paired-end or single-end. Experiments marked as 35x correspond to sequencing depths of 35x. Experiments (EXP) 1–12 are divided into three groups: 1–4, 5–8, and 9–12. Across these groups, $\mu_{FL}$ varies, while $C_{F}$ and $C_{R}$ vary within each group.

## 2 Methods

### 2.1 Simulator pipeline

In this study, we present stLFR-sim, a Python-based simulator designed to generate linked-read sequencing data that mimics the output of the stLFR platform and Illumina sequencers. While it draws partial inspiration from the methodology of LRTK-sim (Zhang *et al.* 2019), stLFR-sim is an original tool that introduces several novel features. Compared to LRTK-sim which is a linked-read simulator for 10X Chromium System, stLFR-sim is optimized for stLFR linked-reads simulation. Furthermore, stLFR-sim offers the original feature of simulating long single-end barcoded reads. In detail, the simulator reproduces the stLFR linked-read sequencing workflow through four main steps: (1) generating a diploid reference genome, (2) simulating long DNA fragments, (3) assigning barcodes to DNA fragments, and (4) generating barcoded Illumina short reads. This pipeline enables realistic simulation of linked-read data, making stLFR-sim a valuable resource for benchmarking sequencing workflows and downstream analysis tools. Each step is elaborated below to provide a comprehensive overview of the simulation process.

#### 2.1.1. Generating the diploid reference genome

stLFR-sim begins with a sample-specific diploid genome as input. While the original LRTK-sim pipeline generated this by inserting phased, high-confidence variants into a reference genome, our approach instead utilizes a phased, realistic diploid assembly from the HG002 sample (see section titled “T2T Realistic Simulation” for details). This diploid reference serves as the foundation for generating synthetic linked-read data, allowing the simulation to capture the genomic complexity of a real individual.

#### 2.1.2. Simulating long DNA fragments

With the diploid genome defined, stLFR-sim proceeds to simulate long DNA fragments. The physical coverage, denoted $C_{F}$, represents the cumulative coverage of all DNA fragments across the genome. The total amount of DNA, *V*, is calculated as $V=C_{F}\times L$, where *L* is the length of the reference genome. To model both haplotypes equally, each is assigned half of the total physical coverage, i.e., $C_{F}/2$. Fragment start positions are randomly distributed across the genome, and fragment lengths are drawn from an exponential distribution with a mean of $\mu_{FL}$. This step ensures that the simulated fragment size distribution closely mirrors that observed in real linked-read sequencing data.

#### 2.1.3. Barcoding DNA fragments

In this step, stLFR-sim emulates the barcoding process used in stLFR technology. There are originally 1536 unique 10-mer barcodes in the stLFR barcode list. stLFR-sim randomly selects 3 barcodes from the list, thereby generating up to 3.62 billion unique 30-mer barcode sequences for labeling DNA fragments. This number (3.62 billion) represents the theoretical maximum number of barcodes; however, the actual number utilized depends on the values of $C_{F}$ and $\mu_{FL}$, and is typically much lower than the maximum. Specifically, the actual number of barcodes can be estimated as $N_{bc}$ = $C_{F}$  × genome size/$\mu_{FL}$. stLFR-sim generates “pseudo” partitions, each assigned a unique barcode.

In contrast to LRTK-sim’s pipeline, which simulates the stochastic barcoding process by assigning multiple fragments to a single partition, stLFR-sim reflects the design of stLFR technology, which enables precise assignment of one fragment per partition. Consequently, each DNA fragment in stLFR-sim is assigned to a distinct partition with a unique barcode, accurately modeling the one-fragment-per-barcode characteristic of stLFR.

#### 2.1.4. Simulating barcoded illumina short reads

The final step in stLFR-sim involves generating barcoded Illumina short reads to cover the simulated long DNA fragments. The total number of reads is calculated as $\frac{C_{R}\times V}{R_{L}}$, where $C_{R}$ denotes the desired read coverage, *V* is the total DNA volume, and $R_{L}$ is the read length, typically set to 150 bp by default. Reads are uniformly distributed across each fragment to mimic even sequencing coverage.

Paired-end short reads are generated with insert sizes sampled from a normal distribution centered at a user-defined mean $\mu_{IS}$. Short reads are tagged with barcodes derived from their associated DNA fragments. To further enhance realism, stLFR-sim incorporates empirical base quality profiles derived from linked-read datasets sequenced on an Illumina HiSeq X platform. Sequencing errors are introduced at random positions based on these quality scores, providing a more accurate simulation of real-world sequencing data. stLFR-sim supports two configurable sequencing error models: a uniform model, in which the per-base error probability (1% by default) remains constant across the read, and a quadratic model, in which the error probability increases progressively with sequencing cycle to better reflect the deterioration of sequencing quality toward the end of longer reads. In the quadratic error model, for a read of length *L*, the error probability at position *i* is defined as


(1) $$p_{i}=p_{\text{start}}+\left(p_{\text{end}}-p_{\text{start}}\right){\left(\frac{i-1}{L-1}\right)}^{2}$$

where $p_{i}$ is the probability of a sequencing error at base *i*, and $p_{\text{start}}$ and $p_{\text{end}}$ represent the error probabilities at the beginning and end of the read, respectively. The normalized position term ranges from 0 to 1, while squaring this term causes the error probability to increase gradually at early sequencing cycles and more rapidly toward the end of the read. Sequencing errors are then independently introduced at each position according to the corresponding probability $p_{i}$. This model provides a simple approximation of the cycle-dependent decline in sequencing accuracy observed for long Illumina reads.

#### 2.1.5. Simulating other sequencing data types

To broaden the scope of the simulation, stLFR-sim also supports the generation of standard Illumina short reads (without barcodes) and long single-end barcoded reads. For simulating unbarcoded Illumina short reads, the barcoding step is simply disabled while the rest of the pipeline remains unchanged. To simulate long single-end barcoded reads, the paired-end sequencing module is replaced with a single-end mode, which randomly samples fixed-length reads from each fragment.

In summary, stLFR-sim provides several advantages over existing linked-read simulators. First, it allows users to flexibly configure key parameters, including $C_{F}$, $C_{R}$, $\mu_{FL}$, $R_{L}$, and $\mu_{IS}$, to accurately model specific experimental conditions. Second, we have extended its functionality to support the simulation of standard Illumina short reads (without barcodes) and long single-end barcoded reads, enhancing its versatility. Finally, stLFR-sim is a lightweight, all-in-one Python package that requires no third-party software. In contrast to simulators like LRSIM, which depend on multiple external tools, stLFR-sim is self-contained and capable of simulating multiple libraries in a single run, making it both efficient and user-friendly for exploring a wide range of experimental designs.

### 2.2 T2T realistic simulation

To simulate reads sequenced within realistic genomic contexts, we utilized the phased diploid assembly of HG002 released by the Human Pangenome Reference Consortium (HPRC) (Liao *et al.* 2023). The paternal and maternal haplotype assemblies were independently aligned to the T2T-CHM13(v2)0.0 reference using minimap2. Contigs from each haplotype that aligned to the target chromosome (e.g. chromosome 6) were then extracted using SAMtools and used as input for our simulation.

minimap2—secondary = no -a—eqx -Y -x asm20 -s 200000-z 10000,50 -r 50,000—end-bonus = 100 -O 5,56 -E 4,1 -B 5 ${ref}$ {asm} | samtools sort -o ${bam}

### 2.3 Simulation configurations

Read libraries were simulated under various configurations to systematically investigate the impact of different parameter combinations. PE100_stLFR libraries consist of paired-end reads with a length of 100 bp, while SE500_stLFR and SE1000_stLFR libraries contain single-end barcoded reads with lengths of 500 bp and 1000 bp, respectively. A total of 12 simulation experiment (EXP1-EXP12) were conducted for each library type. These experiments were categorized into three groups: EXP1-EXP4, EXP5-EXP8, and EXP9-EXP12. Within each group, the parameters $C_{F}$ and $C_{R}$ were varied, while the parameter $\mu_{FL}$ differed between groups. All groups shared consistent values for insertion size, error rate, and number of fragments per barcode ($N_{FL}$).

More specifically, the $C_{F}$-$C_{R}$ combinations ranged from 350–0.1 to 47–0.75 within each group. The $\mu_{FL}$ values were set to 50 kb, 75 kb, and 100 kb for EXP1-EXP4, EXP5-EXP8, and EXP9-EXP12, respectively. Across all experiments, the insertion size, error rate, and number of fragments per barcode ($N_{FL}$) were consistently set to 600 bp, 1%, and 1, respectively. Full simulation configurations are summarized in Table 1.

### 2.4 Aquila_stLFR (v2) and SV calling

We developed Aquila_stLFR (v2) as an updated pipeline for structural variant (SV) calling, based on the original Aquila_stLFR framework (Liu *et al.* 2021). Compared to the previous version, it offers the new feature of processing long single-end barcoded reads while having more optimized software structure. This version is a reference-assisted tool designed to perform local *de novo* assembly of stLFR linked-read data. It reconstructs haplotype-specific blocks using barcode-tagged long fragment reads (LFRs) and performs localized assembly within small genomic regions to achieve high contiguity and accurate SV detection.

Aquila_stLFR (v2) processes stLFR data in three main steps: (1) Haplotype phasing: Long fragment reads (LFRs) are reconstructed and partitioned into haplotype-specific blocks using barcode information and heterozygous SNPs. (2) Local assembly: Within 100 kb genomic chunks, reads are locally assembled using SPAdes. Resulting mini-contigs are then iteratively merged to form full-length haplotype-resolved contigs. (3) SV detection: Structural variants are identified by aligning haploid contigs to the reference genome using Minimap2 (Li 2018), followed by a custom SV detection pipeline based on VolcanoSV-vc (Luo *et al.* 2024).

While steps 1 and 2 remain unchanged from the original Aquila_stLFR, step 3 has been enhanced in version 2 to incorporate the VolcanoSV-vc methodology for more robust SV detection.

VolcanoSV-vc is the variant calling module of VolcanoSV (Luo *et al.* 2024), originally designed for long-read sequencing data. For our linked-reads application, we adapted VolcanoSV-vc by disabling the long-read-specific signature scanning module. In our workflow, VolcanoSV-vc detects SVs from haplotype-resolved contigs aligned to the reference genome using Minimap2. SVs, such as large insertion and deletion SVs, are identified based on CIGAR string information and split alignment signatures.

SV signatures are collected independently for each haplotype, clustered using a nearest-neighbor algorithm, and refined to eliminate redundancy. Within each cluster, the longest signature is retained as the final SV call. Genotypes are then assigned based on the presence of SVs across haplotypes: “1|1” if present in both, and “0|1” or “1|0” if present in only one. Despite potential assembly artifacts, VolcanoSV-vc uses empirically optimized thresholds to ensure accurate clustering and genotyping in the linked-reads context.

The following commands were used to generate the SV calls.

Aquila_stLFR2_step1.py\

-fastq_file ${reads_fastq} \

-bam_file ${bamfile} \

-vcf_file ${SNP_vcf} \

-sample_name HG002 \

-out_dir ${outdir} \

-uniq_map_dir ./Aquila_stLFR/Uniqness_map_hg19 \

-chr_start ${chr_num} -chr_end ${chr_num} -num_threads $t -r $dtype

Aquila_stLFR2_step2.py\

-out_dir ${outdir} \

-num_threads $t \

-reference $reference \

-chr_start $chr_num -chr_end ${chr_num} -r $dtype

Aquila_stLFR2_step3.py\

-contig ${outdir}/Assembly_Contigs_files/Aquila_Contig_chr${chr_num}.fasta \

-ref ${reference} \

-o ${outdir}/SV_ouput/ \

-chr ${chr_num} -t $t

### 2.5 SNP and small INDEL calling

For SNP and INDEL calling, simulated reads were first aligned to the reference genome using BWA-MEM (v0.7.17) (Li and Durbin 2009) for long single-end barcoded reads, and EMA (v0.6.2) (Shajii 2018) for stLFR paired-end reads. Read group information was then added to the alignments using Picard. Finally, SNP and INDEL calling was performed using the GATK (v4.3.0) pipeline (McKenna *et al.* 2010).

The following commands were used to generate the SNP and INDEL calls:

bwa mem\

${reference} \

.${R1_fastq} \

${R2_fastq} \

-t 30 |samtools sort -o ${bwa_bam}

ema align\

-1 ${R1_fastq} \

-2 ${R2_fastq} \

-r ${reference} -t 30 |samtools sort -@ 30 -o ${ema_bam}

java -jar picard.jar AddOrReplaceReadGroups\

I=${raw_bamfile} \

O=${bamfile} \

RGID = 4 \

RGLB = lib1 \

RGPL = ILLUMINA \

RGPU = unit1 \

RGSM = 20

gatk HaplotypeCaller -I $bamfile -O gatk.vcf -R ${reference}

### 2.6 Benchmarking

For SV detection evaluation, we used Truvari (v4.0.0) (English *et al.* 2022) to benchmark our SV callsets against the Genome in a Bottle (GIAB) HG002 SV Tier1 v0.6 high-confidence truth set (Zook *et al.* 2020) on the hg19 reference genome. Truvari was run with a maximum reference distance of 500 bp (*refdist*), a minimum allele sequence similarity of 50% (*pctsim*), a minimum allele size similarity of 50% (*pctsize*), a minimum reciprocal overlap of 1% (*pctovl*), and restrictions to high-confidence regions (*includebed*) and PASS variants (*passonly*). All other parameters were left at their default values.

To contextualize the performance of our method, we additionally compared it with representative approaches from four major paradigms of SV analysis: conventional short-read SV calling, Illumina Complete Long Reads-based SV calling, pangenome-based short-read genotyping, and long-read–based SV detection. Specifically, Manta (v1.6.0) (Chen *et al.* 2016) was used as a representative short-read SV caller, PanGenie (v4.2.1) (Ebler *et al.* 2022) as a pangenome-based short-read genotyper, and VolcanoSV (Luo *et al.* 2024) as a long-read–based SV caller. Manta and PanGenie were both applied to the same Illumina short-read dataset. For PanGenie, genotyping was performed using a pangenome graph constructed from nine Human Pangenome Reference Consortium (HPRC) samples: HG01109, HG01243, HG02055, HG02080, HG02109, HG02145, HG02723, HG03098, and HG03492. VolcanoSV was applied to PacBio HiFi reads. Unless otherwise noted, each method was run using its recommended workflow and standard settings, and the resulting callsets were benchmarked within the same Truvari-based evaluation framework.

SNP and small INDEL benchmarking was performed using hap.py against the NIST v4.2.1 HG002 GRCh37 call set on the hg19 reference genome. Performance was assessed in both high-confidence and difficult genomic regions, including the major histocompatibility complex (MHC), tandem and homopolymer repeats (tandemRep), segmental duplications (segdup), and low-mappability regions (lowmap), using the corresponding BED files from the NIST v4.2.1 genome stratifications. Default parameters were used for all hap.py evaluations.

## 3 Results

Our goal was to explore the potential of linked reads for structural variant (SV) detection and to investigate how sequencing configurations influence SV calling performance. We were also interested in evaluating the potential of long single-end barcoded reads. Therefore, using the simulator stLFR-sim and a high-quality diploid assembly of sample HG002 as the reference genome, we simulated a series of stLFR linked-read datasets, including PE100_stLFR, SE500_stLFR, and SE1000_stLFR. In stLFR-sim, multiple sequencing parameters are incorporated to generate realistic simulations, including $C_{F}$ (coverage of long fragments), $C_{R}$ (coverage of short reads on each long fragment), $\mu_{FL}$ (average fragment length), and sequencing error rate. By tuning these parameters, we were able to model a range of sequencing scenarios observed in real-world experiments. For each sequencing type, we designed 12 parameter configurations, which are detailed in Table 1. For each simulated paired-end library, reads were aligned using the barcode-aware aligner EMA (v0.6.2) (Shajii 2018). For each simulated single-end library, reads were aligned using the aligner BWA-MEM (v0.7.17) (Li and Durbin 2009). SNPs were called using GATK (v4.3.0) (McKenna *et al.* 2010), and SVs were detected using Aquila_stLFR (v2) (Liu *et al.* 2021). The resulting SV calls were then compared to the GIAB SV benchmark set (Li *et al.* 2018) using the benchmarking tool Truvari (v4.0.0) (English *et al.* 2022), generating a comprehensive set of evaluation metrics. We further compared the SV calling performance of long single-end barcoded reads (analyzed using Aquila_stLFR (v2)) with that of traditional paired-end short reads (analyzed using Manta (v1.6.0) (Chen *et al.* 2016) and PanGenie (v4.2.1) (Ebler *et al.* 2022)) and long reads (analyzed using VolcanoSV (Luo *et al.* 2024)), demonstrating the strong potential of long single-end barcoded reads for accurate SV detection. Detailed analyses of these results are presented in the following sections.

### 3.1 Simulated PE100_stLFR data reproduces qualitative SV-calling trends and small-variant performance

In this session, we compared the sequencing configuration and variant calling performance between our simulated data and real data. In our simulation experiment, we simulated PE100_stLFR data across 12 different simulation configurations (Table 1). In our simulation pipeline, the determining parameters were $C_{F}$ (coverage of long fragment), $C_{R}$ (coverage of short reads on each long fragment), $\mu_{FL}$ (average fragment length), and sequencing error rate. Specifically, we simulated 12 different libraries for PE100_stLFR, among which $C_{F}$ ranged from 47–350, $C_{R}$ ranged from 0.1–0.75, $\mu_{FL}$ ranged from 50–100 kb. The error rate was set to 1% for all 12 libraries. For the real PE100_stLFR data, $C_{F}$ was 931, $C_{R}$ was 0.11, $\mu_{FL}$ was 13.9 kb, and the error rate was 1%.

**Figure 1:**
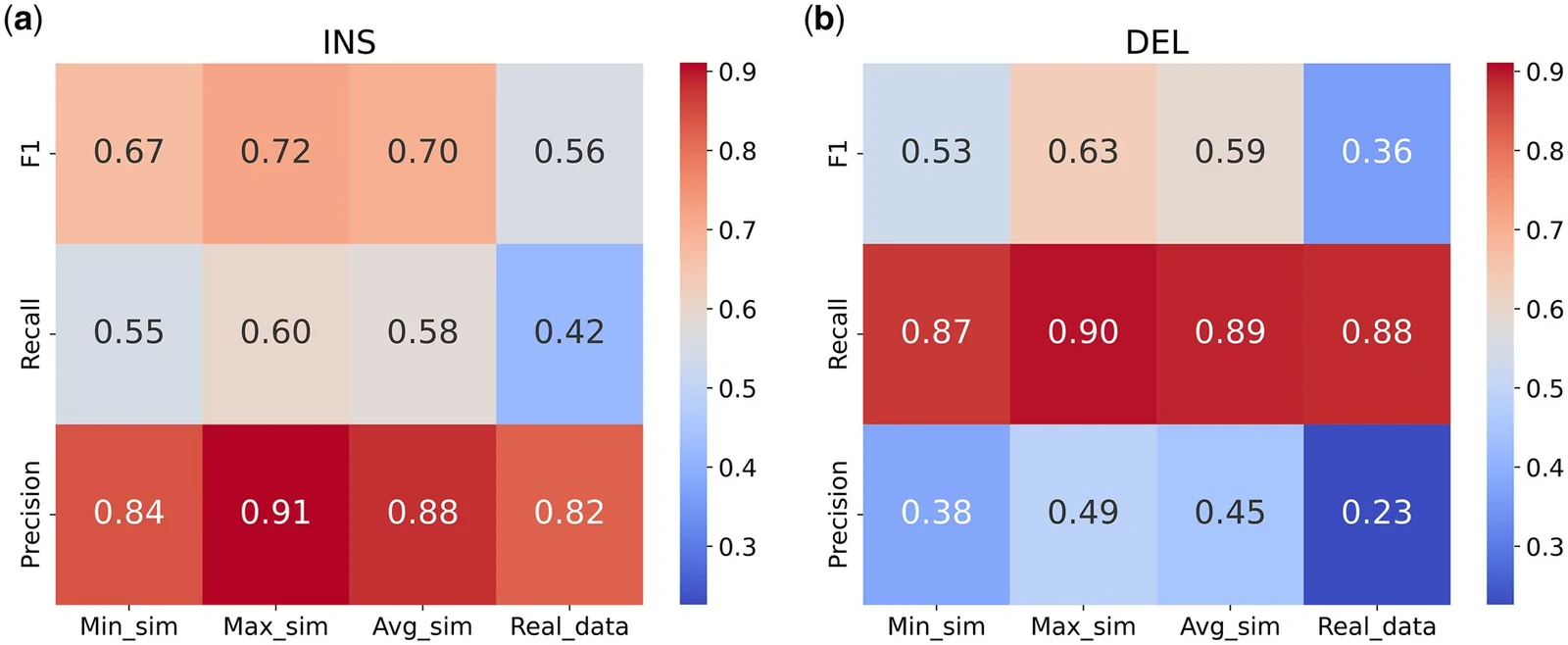
Simulated and real PE100_stLFR SV calling performance comparison *Truvari* (v4.0.0) was applied to benchmark the SV callset against the GIAB SV benchmark set. Across 12 different simulation configurations, the minimum (Min_sim), maximum (Max_sim) and average (Avg_sim) value of F1, recall and precision score were calculated. (a) F1, recall, precision for insertion SV calling. (b) F1, recall, precision for deletion SV calling.

For SV calling, we first compared the overall simulated data across experiments and real PE100_stLFR data (Fig. 1). We also compared the performance between simulated experiment 1 of PE100_stLFR and real PE100_stLFR (Table 2) since their sequencing configurations were most similar. From both analyses, the simulated and real PE100_stLFR showed an overall similar trend and accuracy: for insertion SV, the precision was high but the recall was relatively lower, while for deletion SVs, the recall was high but the precision was generally lower. A key objective of our simulation was to assess the potential performance gains of stLFR reads under configurations optimized for structural variant (SV) detection. To this end, we simulated fragment lengths ranging from 50 to 100 kb, exceeding the typical fragment lengths observed in real datasets (e.g. the real dataset used in this study had an average fragment length of 13.9 kb). As a result, the simulated PE100_stLFR dataset achieved a higher average F1 score than the real PE100_stLFR data, consistent with the expectation that longer fragments enhance SV-calling performance.

**Table 2:** Comparison of structural variant calling performance for deletion and insertion SVs between PE100_stLFR (EXP1) and PE100_stLFR (real).

INS	TP	FP	FN	precision	recall	f1	TP-gt	FP-gt	gt_concordance
PE100_stLFR (EXP1)	174	26	136	0.87	0.56	0.68	134	40	0.77
PE100_stLFR (real)	130	28	180	0.82	0.42	0.56	112	18	0.86
**DEL**	**TP**	**FP**	**FN**	**precision**	**recall**	**f1**	**TP-gt**	**FP-gt**	**gt_concordance**
PE100_stLFR (EXP1)	233	381	27	0.38	0.90	0.53	210	23	0.90
PE100_stLFR (real)	230	788	30	0.23	0.88	0.36	208	22	0.90

Performance is summarized using true positives (TP), false positives (FP), false negatives (FN), precision, recall, F1 score, genotype true positives (TP-gt), genotype false positives (FP-gt), and genotype concordance.

**Figure 2:**
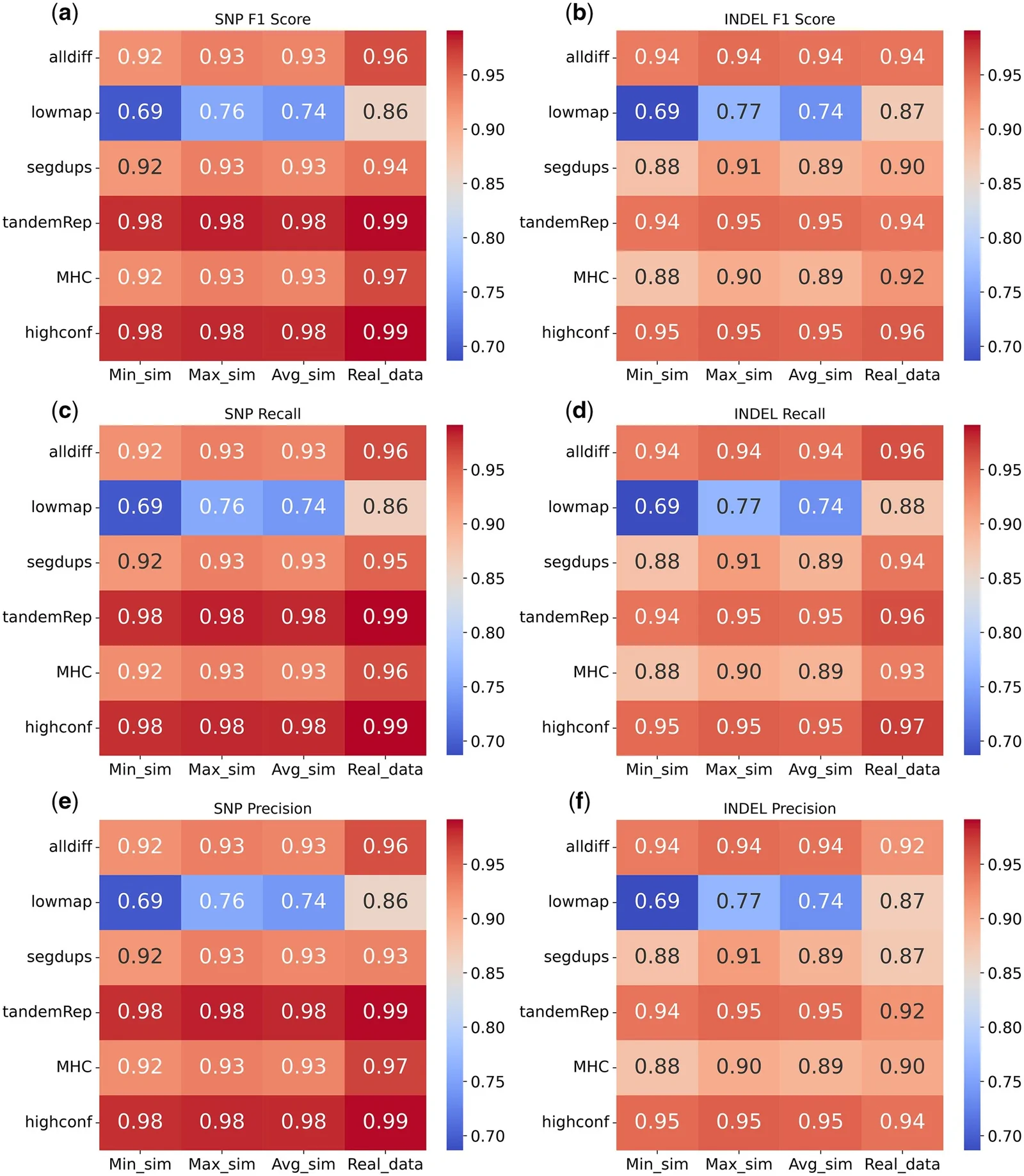
Simulated and real PE100_stLFR SNP INDEL calling performance comparison *hap*.*py* v0.3.15 was applied to benchmark the SNP and small INDEL callset against the NIST v4.2.1 call set. Across 12 different simulation configurations, the minimum (Min_sim), maximum (Max_sim) and average (Avg_sim) value of F1, recall and precision score were calculated. The evaluation was applied to different regions on the genome, including all difficult regions (alldiff), low mappability regions (lowmap), segmental duplication regions (segdups), tandem repeat regions (tandemRep), major histocompatibility complex regions (MHC) and high confidence region defined by GIAB for HG002 (highconf). (a) F1 for SNP calling. (b) F1 for INDEL calling. (c) Recall for SNP calling. (d) Recall for small INDEL calling. (e) Precision for SNP calling. (f) Precision for small INDEL calling.

We also compared SNP, small INDEL calling performance between simulated and real PE100_stLFR data. For SNP and small INDEL calling (Fig. 2), we evaluated performance across 12 different simulation configurations. Across each configuration, the minimum (Min_sim), maximum (Max_sim), and average (Avg_sim) values of F1 score, recall, and precision were calculated. The evaluation was performed across multiple genomic regions, including all difficult regions (alldiff), low mappability regions (lowmap), segmental duplication regions (segdups), tandem repeat regions (tandemRep), the major histocompatibility complex (MHC), and the GIAB high-confidence regions for HG002 (highconf). Our simulated data on average showed comparable result to real data. Especially, in the high-confidence region, the difference between average simulation data F1 and real data F1 was only 0.01 for both SNP and small INDEL calling.

Together, these comparisons indicate that stLFR-sim captures several broad characteristics of PE100_stLFR variant-calling performance, including the contrasting precision-recall patterns of insertion and deletion SV calls and the high SNP and small-INDEL accuracy observed in high-confidence regions. However, because the simulated and real libraries were not parameter matched, these results should not be interpreted as demonstrating exact reproduction of real-data performance. We therefore used the simulator primarily to compare sequencing configurations under controlled in-silico conditions.

### 3.2 Long single-end barcoded reads harbor strong potential for SV calling

In this section, we evaluated the performance of SV calling across three types of linked-reads libraries: PE100_stLFR, SE500_stLFR, and SE1000_stLFR. Each was sequenced at 35x coverage. For each library type, we analyzed 12 datasets to investigate the effects of sequencing parameters on SV calling. SVs were identified using Aquila_stLFR (v2), an extension of the previous linked-read-based SV detection tool Aquila_stLFR (Liu *et al.* 2021). We benchmarked the resulting call sets against the GIAB SV benchmark set using Truvari (v4.0.0) (English *et al.* 2022) with the following parameters: p = 0.5, *P *= 0.5, *r *= 500, *O *= 0.01. Our evaluation focused on insertion SVs (INSs) and deletion SVs (DELs) larger than 50 bp within the high-confidence regions defined by GIAB.

**Figure 3:**
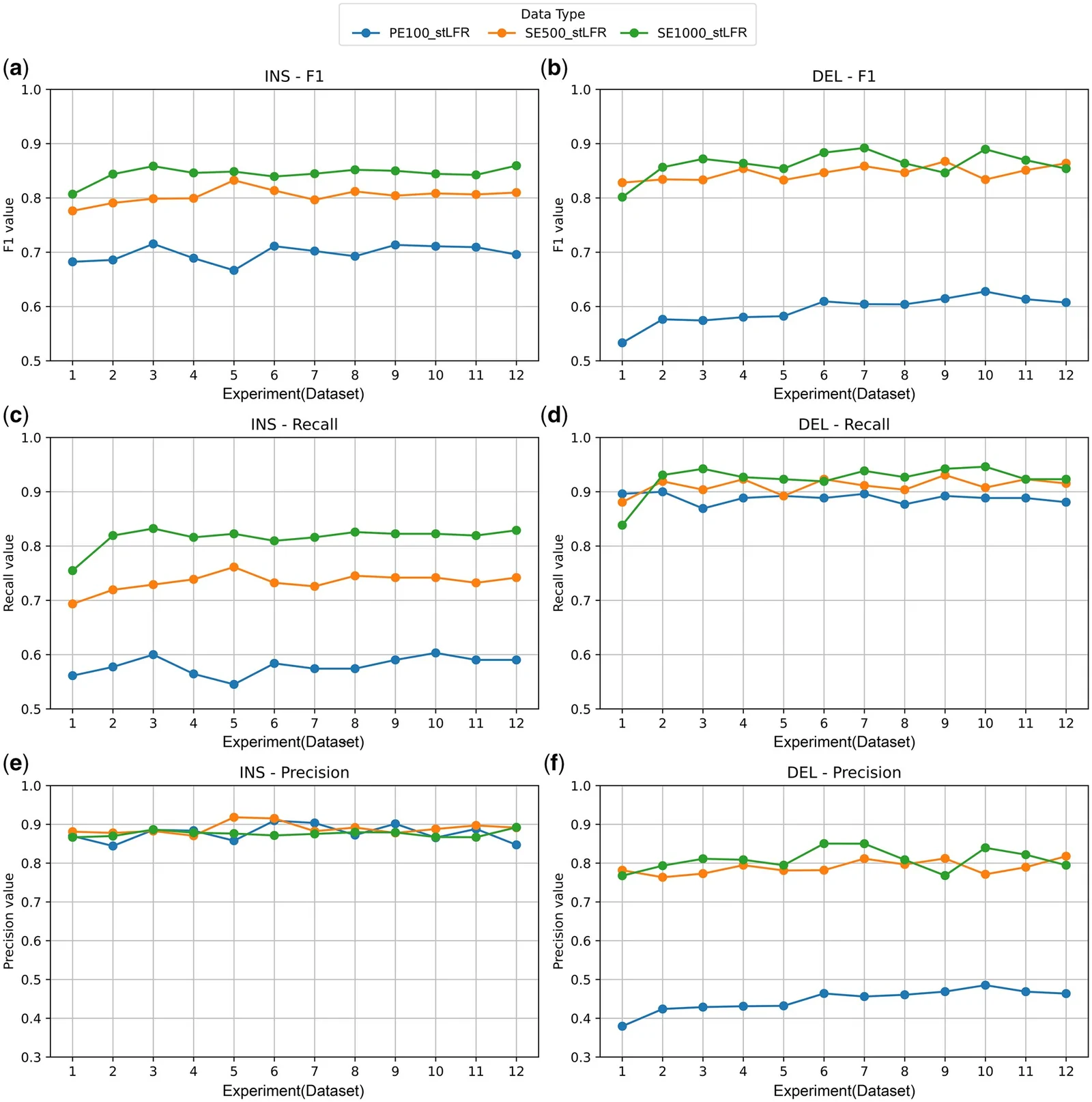
Structural variant detection performance across different single libraries. Aquila_stLFR (v2) was applied to generate the SV callsets. Truvari (v4.0.0) was used to benchmark the result against GIAB SV benchmark set (parameters: p = 0.5, *P *= 0.5, *r *= 500, *O *= 0.01). Libraries are color-coded as follows: blue for PE100_stLFR, orange for SE500_stLFR, and green for SE1000_stLFR. (a, c, e) Show the F1 score, recall, and precision for insertion SV (INS) calls (b, d, f) show the F1 score, recall, and precision for deletion SV (DEL) calls.

For insertion SVs, the performance of the three library types varied significantly across the evaluated metrics, as shown in Fig. 3a, c, and e. SE1000_stLFR demonstrated the highest F1 score, ranging from 0.81 to 0.86 with an average of 0.84 across all 12 datasets, indicating a superior balance between precision and recall. SE500_stLFR followed closely with F1 scores ranging from 0.78 to 0.83 (average 0.80), while PE100_stLFR exhibited the lowest performance, with F1 scores between 0.67 to 0.72 (average 0.70). For recall, SE1000_stLFR again outperformed the others, achieving values between 0.75 and 0.83 (average 0.82), reflecting its ability to detect true insertion SVs. SE500_stLFR showed moderate recall, ranging from 0.69 to 0.76 (average 0.73), whereas PE100_stLFR performed the worst, with recall values between 0.55 and 0.60 (average 0.58), highlighting its limitations in sensitivity. In contrast, precision was highest in SE500_stLFR, ranging from 0.87 to 0.92 with an average of 0.89, reflecting its strong accuracy in identifying true positives. PE100_stLFR followed closely, with a precision range of 0.84–0.91 (average 0.88), while SE1000_stLFR, despite its overall superior performance, showed a slightly narrower range of 0.87–0.89 (average 0.88). These results suggested that longer read lengths, as in SE1000_stLFR, enhanced recall but incurred a modest trade-off in precision, whereas shorter reads, such as PE100_stLFR, prioritized precision but struggled with recall.

For deletion SVs, SE1000_stLFR consistently outperformed other libraries across most metrics (Fig. 3b, d, and f). SE1000_stLFR achieved F1 scores ranging from 0.80 to 0.89, with an average of 0.86, indicating strong overall performance. SE500_stLFR was closely competitive, with F1 scores between 0.83 and 0.87 (average 0.85), while PE100_stLFR lagged behind, ranging from 0.53 to 0.63 (average 0.59), reflecting reduced balance between precision and recall. In terms of recall, all three datasets performed relatively well: SE1000_stLFR ranged from 0.84 to 0.95 (average 0.92), SE500_stLFR from 0.88 to 0.93 (average 0.91), and PE100_stLFR from 0.87 to 0.90 (average 0.89). These results collectively highlighted the robustness of DEL detection across datasets, with SE1000_stLFR showing the most consistent and accurate performance. Precision, however, remained a key differentiator. SE1000_stLFR again led, with precision values ranging from 0.77 to 0.85 (average 0.81), followed closely by SE500_stLFR, which ranged from 0.76 to 0.82 (average 0.79). In contrast, PE100_stLFR showed substantially lower precision, with values between 0.38 and 0.49 (average 0.45), highlighting a higher rate of false positives in deletion SV calls. These results emphasized that SE1000_stLFR, benefiting from longer reads, consistently offered the best trade-off between precision and recall. Conversely, the shorter reads of PE100_stLFR appeared to hinder accurate deletion SV detection, particularly in maintaining precision—likely due to the challenges of resolving complex genomic regions with limited sequence context.

Overall, insertion SV calls favored high precision but lower recall, while deletion SV calls showed higher recall at the cost of precision, emphasizing distinct trade-offs between the two SV types. Longer reads were more effective in balancing these metrics, particularly for deletion SV detection. In contrast, shorter reads such as PE100_stLFR struggled, exhibiting reduced recall for insertion SVs and diminished precision for deletion SVs.

### 3.3 Long single-end barcoded reads achieve competitive SV calling performance across sequencing strategies

Given the substantial improvement of SE1000_stLFR over PE100_stLFR in the preceding analyses, we next investigated how SE1000_stLFR compared with a broader range of sequencing-based strategies for structural variant detection, particularly long-read approaches that have shown strong performance in SV discovery. To address this, we selected SE1000_stLFR EXP5-8, which share a mean fragment length of 75 kb while spanning four combinations of physical coverage ($C_{F}$) and short-read coverage per long fragment ($C_{R}$), and benchmarked all four configurations across the HG002 whole genome against representative methods spanning conventional short-read SV calling, synthetic long-read-based SV calling, pangenome graph-based short-read genotyping, and long-read-based SV calling. Specifically, we included Manta (Chen *et al.* 2016) as a representative short-read SV caller, Illumina Complete Long Reads (ICLR) and Sniffles2 (Smolka *et al.* 2024) as synthetic long-read-based SV calling pipeline, PanGenie (Ebler *et al.* 2022) as a pangenome-based short-read genotyping approach, and VolcanoSV (Luo *et al.* 2024) as a long-read-based SV calling benchmark. VolcanoSV was applied to PacBio HiFi data, whereas PanGenie genotyped Illumina short reads against a pangenome graph built from nine diploid high-quality assemblies. The relevant real data are provided in the Data Availability section. This comprehensive and stratified comparison enabled us to evaluate SE1000 stLFR across a spectrum of SV calling strategies, ranging from conventional short-read analysis to pangenome graph-assisted and long-read-enabled frameworks. Because these approaches differ in both sequencing data type and analytical strategy, the benchmark was intended to provide comparative performance context across representative SV analysis paradigms rather than a controlled head-to-head comparison of sequencing technologies.

As summarized in Table 3, SE1000_stLFR showed consistently strong performance for both insertion and deletion SVs. Overall, it substantially outperformed the conventional short-read caller Manta, achieved performance comparable to the pangenome-based genotyper PanGenie and the ICLR-based workflow, and approached that of VolcanoSV, which remained the strongest method overall. For insertion SVs, SE1000_stLFR achieved a substantially better balance between precision and recall than both Manta and ICLR_Sniffles2, with markedly higher F1 scores and recall. It also outperformed PanGenie in overall detection accuracy, although VolcanoSV retained the best performance. For deletion SVs, SE1000_stLFR again clearly exceeded Manta and achieved an F1 score identical to that of PanGenie and close to that of ICLR_Sniffles2, while trailing VolcanoSV only modestly. Genotyping concordance showed a similarly strong overall pattern. For insertion SVs, SE1000_stLFR substantially outperformed ICLR_Sniffles2 and PanGenie, whereas for deletion SVs, it achieved markedly higher genotype concordance than ICLR_Sniffles2 and a level comparable to PanGenie, although it remained below Manta and VolcanoSV. Together, these results indicate that SE1000_stLFR markedly advances beyond conventional short-read SV calling and can achieve performance competitive with ICLR, pangenome-, and long-read-based approaches. These findings suggest that extended-length single-end barcoded sequencing could represent a promising intermediate strategy between standard short-read and long-read technologies for structural variant analysis.

**Table 3:** Comparison of structural variant calling performance for deletion and insertion SVs on whole genome HG002 among SE1000_stLFR, Manta, ICLR_Sniffles2, PanGenie and VolcanoSV.

INS	TP	FP	FN	precision	recall	F1	TP-gt	FP-gt	gt_concordance
SE1000_stLFR (EXP5)	4229	535	1052	0.89	0.80	0.84	3908	321	0.92
SE1000_stLFR (EXP6)	4216	555	1065	0.88	0.80	0.84	3903	313	0.93
SE1000_stLFR (EXP7)	4241	552	1040	0.88	0.80	0.84	3909	332	0.92
SE1000_stLFR (EXP8)	4220	545	1061	0.89	0.80	0.84	3838	382	0.91
Manta	1957	66	3324	0.97	0.37	0.54	1854	103	0.95
ICLR_Sniffles2	2071	104	3210	0.95	0.39	0.56	900	1171	0.43
PanGenie	4530	1937	751	0.70	0.86	0.77	3488	1042	0.77
VolcanoSV	4964	591	317	0.89	0.94	0.92	4883	81	0.98
**DEL**	**TP**	**FP**	**FN**	**precision**	**recall**	**F1**	**TP-gt**	**FP-gt**	**gt_concordance**
SE1000_stLFR (EXP5)	3777	1226	339	0.75	0.92	0.83	3554	223	0.94
SE1000_stLFR (EXP6)	3804	1012	312	0.79	0.92	0.85	3580	224	0.94
SE1000_stLFR (EXP7)	3820	1025	296	0.79	0.93	0.85	3566	254	0.93
SE1000_stLFR (EXP8)	3796	1011	320	0.79	0.92	0.85	3539	257	0.93
Manta	2617	508	1499	0.84	0.64	0.72	2556	61	0.98
ICLR_Sniffles2	3451	209	665	0.94	0.84	0.89	2706	745	0.78
PanGenie	3576	718	540	0.83	0.87	0.85	3378	198	0.94
VolcanoSV	3895	261	221	0.94	0.95	0.94	3859	36	0.99

Performance is summarized using true positives (TP), false positives (FP), false negatives (FN), precision, recall, F1 score, genotype true positives (TP-gt), genotype false positives (FP-gt), and genotype concordance. For insertion detection, SE1000_stLFR substantially outperformed both the short-read caller Manta and Sniffles2 applied to ICLR. For deletion detection, SE1000_stLFR also substantially outperformed Manta, while achieving an F1 score comparable to ICLR_Sniffles2 and higher genotype concordance. Across both deletion and insertion SV detection, SE1000_stLFR achieved competitive performance relative to the long-read-based caller VolcanoSV and the pangenome-based genotyper PanGenie, indicating that 1000-bp single-end barcoded reads can recover much of the SV calling capability typically associated with long-read approaches.

### 3.4 Error stratification reveals shared genomic challenges and size-dependent limitations

**Figure 4:**
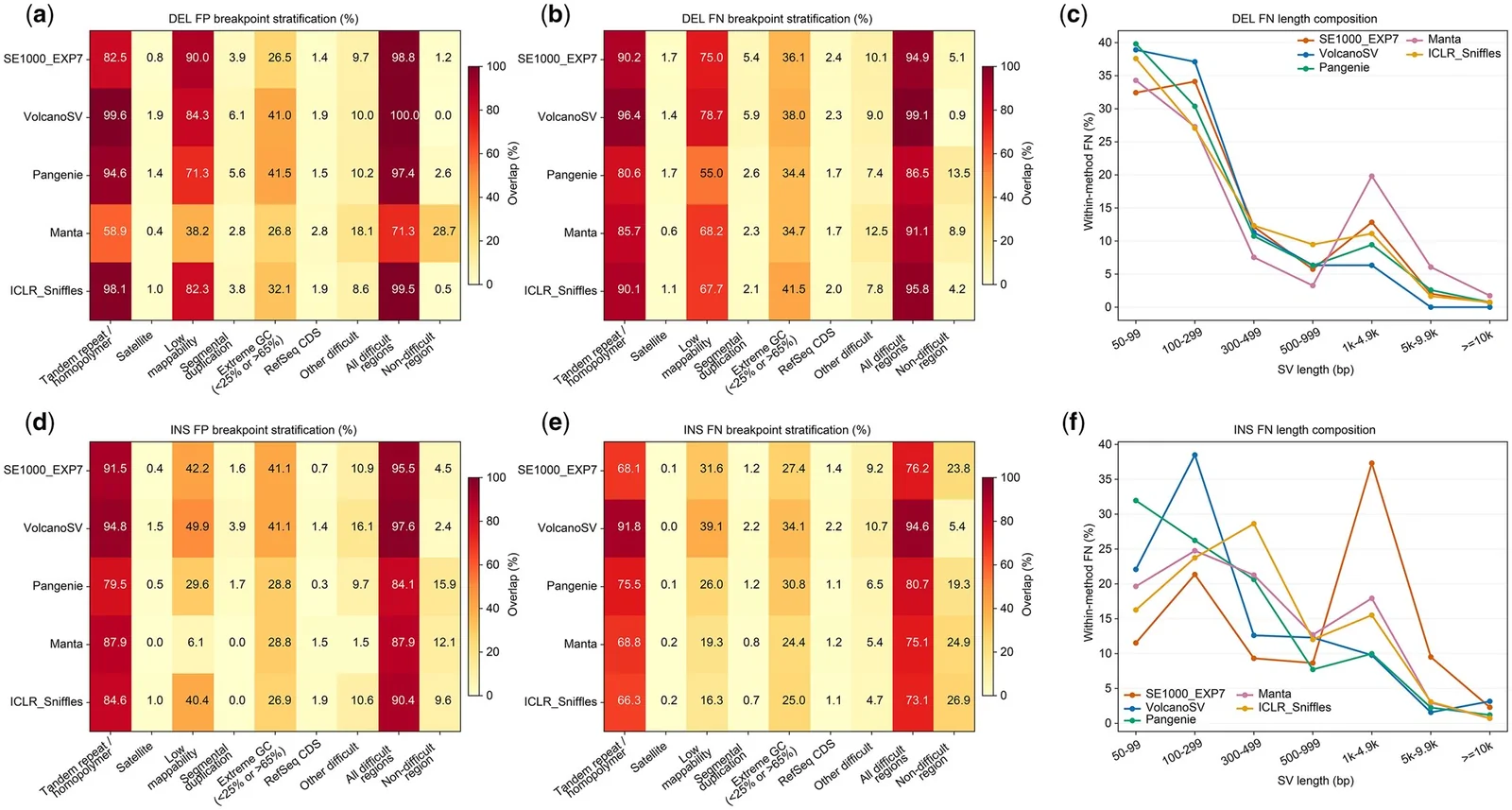
Stratification of SV errors by genomic context and size. False-positive (FP) and false-negative (FN) SVs from SE1000_stLFR and representative SV calling pipelines were stratified by genomic context and variant size. (a, b) Genomic-context distribution of deletion FPs and FNs, including tandem repeats/homopolymers, satellite regions, low-mappability regions, segmental duplications, regions with extreme GC content, RefSeq coding regions, and other difficult regions. (c) Size distribution of deletion FNs. (d, e) Genomic-context distribution of insertion FPs and FNs. (f) Size distribution of insertion FNs. Across methods, most FP and FN events were concentrated in difficult genomic regions. SE1000_stLFR showed error patterns broadly consistent with those of the other pipelines, although its insertion FNs showed a relatively higher contribution from the 1–4.9 kb size range.

To further investigate the practical limitations of SE1000_stLFR, we stratified false-positive (FP) and false-negative (FN) SVs by genomic context and variant size and compared their distributions with those from other SV calling strategies (Fig. 4). For both deletions and insertions, errors from SE1000_stLFR were strongly enriched in difficult genomic regions, broadly consistent with the patterns observed for VolcanoSV, PanGenie, Manta, and ICLR_Sniffles2 (Fig. 4a-b and d-e). In particular, low-mappability regions, tandem repeats/homopolymers, and regions with extreme GC content accounted for substantial fractions of the observed errors. Overall, 98.8% and 94.9% of deletion FPs and FNs from SE1000_stLFR, respectively, overlapped difficult regions, while the corresponding proportions for insertion FPs and FNs were 95.5% and 76.2%. These patterns were broadly consistent with those observed for the other evaluated SV calling strategies, including VolcanoSV, PanGenie, Manta, and ICLR Sniffles2. These results indicate that most SE1000 stLFR errors occur in genomic contexts that are also challenging for other SV calling approaches, although this analysis does not exclude error modes specific to the simulated 1000-bp reads.

We further examined the size distribution of FNs (Fig. 4c and f). Deletion FNs from SE1000_stLFR showed a size profile broadly consistent with those of the other pipelines, with most missed variants concentrated in the smaller SV size ranges and progressively fewer FNs among larger deletions. For insertions, greater variability was observed across methods. SE1000_stLFR showed a relatively pronounced enrichment of FNs in the 1–4.9 kb range, whereas the other pipelines exhibited different size-dependent patterns.

### 3.5 Long single-end barcoded reads tolerate elevated sequencing error rates up to 2% for SV calling

In our previous simulations, long single-end reads were generated using a simplified uniform 1% sequencing error model. In practice, however, sequencing error rates generally increase with sequencing cycle and may therefore vary substantially across longer reads. To further approximate cycle-dependent sequencing errors, we implemented a quadratic error model in which the per-base error probability progressively increases from the beginning to the end of each read. Using the best-performing configuration from experiment 7, we then performed an error-rate stress test at average sequencing error rates of 2% and 5%.

As shown in Table S1, SV calling remained relatively robust at an average error rate of 2% compared with the uniform 1% error model. For SE500_stLFR, insertion F1 decreased from 0.81 to 0.75 and deletion F1 decreased from 0.84 to 0.76. For SE1000_stLFR, insertion F1 decreased from 0.84 to 0.82 and deletion F1 decreased from 0.89 to 0.83. This moderate reduction likely reflects the sensitivity of the Aquila_stLFR2 pipeline to sequencing errors, as it relies on SNP calls for haplotype phasing and incorporates a local assembly step, both of which may be affected by increased error rates.

In contrast, SV calling performance deteriorated substantially at an average error rate of 5%. Insertion F1 decreased to 0.04 and 0.17 for SE500_stLFR and SE1000_stLFR, respectively, while deletion F1 decreased to 0.04 and 0.21. To investigate a potential source of this performance loss, we evaluated SNP-calling accuracy because SNP calls are used directly for haplotype phasing in Aquila_stLFR2. Within high-confidence regions, SNP F1 scores at a 5% average error rate decreased to 0.64 for SE500_stLFR and 0.72 for SE1000_stLFR, compared with 0.92 and 0.91, respectively, under the 1% error model. The concurrent reductions in SNP- and SV-calling accuracy suggest that impaired SNP calling, and consequently less accurate haplotype phasing, likely contributes to the degradation of downstream SV detection at high sequencing error rates. Local assembly may represent an additional source of sensitivity, as assemblers such as SPAdes (Bankevich *et al.* 2012) were primarily developed for relatively low-error short-read sequencing data. Together, these results indicate that long single-end barcoded reads can tolerate moderate increases in sequencing error, with SV calling remaining relatively robust at an average error rate of 2%. However, substantially higher error rates may require variant-calling, phasing, and assembly methods specifically optimized for long reads with elevated sequencing error rates.

## 4 Discussion

In this study, we evaluated the capabilities and limitations of long single-end barcoded reads of 500 bp (SE500_stLFR) and 1000 bp (SE1000_stLFR), a conceptual extension of the traditional paired-end linked-read technology (PE100_stLFR), in detecting structural variants (SVs).

In terms of structural variant detection, our analysis underscores the significant impact of read length on SV calling, clearly demonstrating that longer single-end barcoded reads substantially enhance SV detection accuracy compared to shorter paired-end linked-reads. Specifically, SE1000_stLFR consistently provided the optimal balance between precision and recall, enabling robust identification of insertion and deletion SVs greater than 50 bp. SE500_stLFR demonstrated intermediate performance, falling between SE1000_stLFR and PE100_stLFR. While PE100_stLFR reads yielded high precision in insertion SV detection and high recall in deletion SV detection, their limited length restricted the recall in insertion SV detection and precision in deletion SV detection, resulting overall lower accuracy. Further, our analysis revealed that SV discovery using SE1000_stLFR reads approached the performance of the PacBio HiFi-based long-read workflow, although the latter remained superior overall. These findings highlight that increasing read length in linked-read sequencing markedly enhances the resolution of structural complexity, helping to overcome limitations inherent to conventional short-read sequencing methods. Employing longer single-end reads, or strategically integrating them in hybrid approaches, represents a promising direction for improving comprehensive genomic analyses and structural variant detection in future designs of linked-read sequencing libraries and studies.

Despite the promising performance, several limitations of the simulation and real-data comparison should be noted. The simulated PE100_stLFR libraries were not configured to reproduce the exact characteristics of the available real dataset. In particular, the simulated mean fragment lengths ranged from 50 to 100 kb, compared with 13.9 kb in the real dataset, and simulated physical coverage ranged from $C_{F}=47$ to 350, compared with $C_{F}=931$ in the real data. These differences likely contributed to the higher SV-calling F1 scores observed in the simulated datasets. Therefore, the agreement between simulated and real PE100_stLFR data should be interpreted primarily as qualitative agreement in precision–recall trade-off patterns rather than exact agreement in absolute performance.

Importantly, this study was designed as an in silico scoping experiment conducted in collaboration with a sequencing-platform company. Its primary goal was not to reproduce a specific existing stLFR library in every experimental detail, but to explore whether extended-length single-end barcoded reads represent a sufficiently promising design direction to justify further technological development. In this context, simulation provides a practical way to evaluate prospective library configurations before the corresponding sequencing chemistry and workflow are fully established.

Beyond the specific findings presented in this study, we envision stLFR-sim as a broadly useful resource for the genomics community. Rather than requiring costly and time-consuming experimental development to evaluate every potential sequencing strategy, stLFR-sim enables systematic in silico exploration of key sequencing parameters, including read length, fragment length, physical coverage, barcode assignment, and sequencing depth. In addition, it provides a flexible benchmark platform for evaluating downstream computational methods, such as structural variant callers, phasing algorithms, and assembly tools, under diverse sequencing scenarios. As sequencing chemistries continue to evolve, the simulator can readily be adapted to assess future library designs that are not yet technically feasible.

More broadly, this work demonstrates the value of simulation-guided sequencing technology design. This study was conceived as an in-silico scoping experiment with the goal of evaluating whether extended-length single-end barcoded reads represent a sufficiently promising design direction to justify further technological development. Rather than simply showing that longer reads improve structural variant detection, our framework provides a quantitative approach for evaluating how changes in sequencing architecture translate into gains in variant discovery, enabling rational assessment of trade-offs between library design and variant-detection performance. Although the extended-length single-end barcoded reads investigated here remain conceptual, they illustrate how simulation can be used to evaluate prospective sequencing designs before substantial engineering effort is invested. We anticipate that this simulation-driven strategy will facilitate the development of next-generation sequencing technologies and the computational methods designed to analyze them.

## 5 Conclusion

In this study, we systematically investigated the impact of different linked-read sequencing designs on structural variant (SV) detection. Our results show that long single-end barcoded reads, particularly SE1000_stLFR, substantially improve SV detection by achieving a more favorable balance between precision and recall. Overall, this work highlights the potential of long single-end barcoded reads as a powerful extension of existing stLFR technology. Our results demonstrate that extending read length within a barcoded linked-read architecture can substantially improve SV detection performance. If such long single-end barcoded reads become technically feasible, they could provide a promising intermediate strategy between conventional short-read and long-read technologies for SV discovery.

## Supplementary Material

vbag267_Supplementary_Data

## Data Availability

All VCF files supporting the findings of this study are available at https://doi.org/10.5281/zenodo.14338785. The difficult genomic regions analyzed in this study were obtained from the NIST v4.2.1 genome stratifications v3.1 dataset, including all difficult regions (https://ftp-trace.ncbi.nlm.nih.gov/ReferenceSamples/giab/release/genome-stratifications/v3.1/GRCh37/Union/GRCh37_alldifficultregions.bed.gz), low-mappability regions (https://ftp-trace.ncbi.nlm.nih.gov/ReferenceSamples/giab/release/genome-stratifications/v3.1/GRCh37/mappability/GRCh37_lowmappabilityall.bed.gz), segmental duplication regions (https://ftp-trace.ncbi.nlm.nih.gov/ReferenceSamples/giab/release/genome-stratifications/v3.1/GRCh37/SegmentalDuplications/GRCh37_segdups.bed.gz), tandem repeat regions (https://ftp-trace.ncbi.nlm.nih.gov/ReferenceSamples/giab/release/genome-stratifications/v3.1/GRCh37/LowComplexity/GRCh37_AllTandemRepeatsandHomopolymers_slop5.bed.gz), and the MHC region (https://ftp-trace.ncbi.nlm.nih.gov/ReferenceSamples/giab/release/genome-stratifications/v3.1/GRCh37/OtherDifficult/GRCh37_MHC.bed.gz). NIST v4.2.1 HG002 GRCh37 call set is available at https://ftp-trace.ncbi.nlm.nih.gov/ReferenceSamples/giab/release/AshkenazimTrio/HG002_NA24385_son/NISTv4.2.1/GRCh37/ HG002 Illumina sequencing reads used to benchmark PanGenie and Manta were obtained from https://www.ebi.ac.uk/ena/browser/view/PRJEB35491. The assemblies used to build the pangenome graph for PanGenie are available at https://s3-us-west-2.amazonaws.com/human-pangenomics/index.html?prefix=working/HPRC_PLUS/; the specific assembly release used was year1_freeze_assembly_v2. SV calling results generated by VolcanoSV were obtained from https://zenodo.org/records/10456758. The Pacbio HiFi reads used for VolcanoSV SV calling are released on https://ftp-trace.ncbi.nlm.nih.gov/ReferenceSamples/giab/data/AshkenazimTrio/HG002_NA24385_son/PacBio_CCS_15kb_20kb_chemistry2/reads/

## Code availability

stLFR-sim is available at https://github.com/maiziezhoulab/stLFRsim under the MIT License.
