# Supplementary material for: A little longer, a lot better: simulation-guided exploration of extended-length single-end barcoded reads for structural variant detection
Source: Bioinform Adv. 2026 Sep 8;6(1):vbag267. doi: 10.1093/bioadv/vbag267 (PMC13614717; doi:10.1093/bioadv/vbag267)
Supplement: Supplementary file 1 [file vbag267_supplementary_data.pdf]

A little longer, a lot better: simulation-guided exploration of extended-length single-end barcoded reads for structural variant detection

Luo et al.

Contents

1 Supplementary Tables **2**

S1 Error-rate stress test of structural variant calling performance for insertion and deletion SVs using SE500_stLFR and SE1000_stLFR reads 2

1 Supplementary Tables

INS	TP	FP	FN	precision	recall	F1	TP-gt	FP-gt	gt_concordance
SE500_stLFR (1%)	230	29	80	0.89	0.74	0.81	209	21	0.91
SE500_stLFR (2%)	200	22	110	0.90	0.65	0.75	156	44	0.78
SE500_stLFR (5%)	6	2	304	0.75	0.02	0.04	0	6	0.00
SE1000_stLFR (1%)	255	39	55	0.87	0.82	0.84	237	18	0.93
SE1000_stLFR (2%)	242	39	68	0.86	0.78	0.82	212	30	0.88
SE1000_stLFR (5%)	29	5	281	0.85	0.09	0.17	11	18	0.38
DEL	TP	FP	FN	precision	recall	F1	TP-gt	FP-gt	gt_concordance
SE500_stLFR (1%)	236	70	24	0.77	0.91	0.83	209	27	0.89
SE500_stLFR (2%)	211	85	49	0.71	0.81	0.76	162	49	0.77
SE500_stLFR (5%)	6	8	254	0.43	0.02	0.04	1	5	0.17
SE1000_stLFR (1%)	246	47	14	0.84	0.95	0.89	235	11	0.96
SE1000_stLFR (2%)	231	67	29	0.78	0.89	0.83	205	26	0.89
SE1000_stLFR (5%)	33	19	227	0.63	0.13	0.21	13	20	0.39

Table S1: **Error-rate stress test of structural variant calling performance for insertion and deletion SVs using SE500_stLFR and SE1000_stLFR reads.** Performance is summarized using true positives (TP), false positives (FP), false negatives (FN), precision, recall, F1 score, genotype true positives (TP-gt), genotype false positives (FP-gt), and genotype concordance, at simulated sequencing error rates of 1%, 2%, and 5%.
